# Postbiotics against Obesity: Perception and Overview Based on Pre-Clinical and Clinical Studies

**DOI:** 10.3390/ijms24076414

**Published:** 2023-03-29

**Authors:** Seon-Joo Park, Anshul Sharma, Hae-Jeung Lee

**Affiliations:** 1 Department of Food and Nutrition, College of Bionanotechnology, Gachon University, Seongnam-si 13120, Republic of Korea; chris0825@gachon.ac.kr; 2 Institute for Aging and Clinical Nutrition Research, Gachon University, Seongnam-si 13120, Republic of Korea; 3 Department of Health Sciences and Technology, GAIHST, Gachon University, Incheon 21999, Republic of Korea

**Keywords:** postbiotics, paraprobiotics, obesity, lactic acid bacteria, metabolites, probiotics

## Abstract

Overweight and obesity are significant global public health concerns that are increasing in prevalence at an alarming rate. Numerous studies have demonstrated the benefits of probiotics against obesity. Postbiotics are the next generation of probiotics that include bacteria-free extracts and nonviable microorganisms that may be advantageous to the host and are being increasingly preferred over regular probiotics. However, the impact of postbiotics on obesity has not been thoroughly investigated. Therefore, the goal of this review is to gather in-depth data on the ability of postbiotics to combat obesity. Postbiotics have been reported to have significant potential in alleviating obesity. This review comprehensively discusses the anti-obesity effects of postbiotics in cellular, animal, and clinical studies. Postbiotics exert anti-obesity effects via multiple mechanisms, with the major mechanisms including increased energy expenditure, reduced adipogenesis and adipocyte differentiation, suppression of food intake, inhibition of lipid absorption, regulation of lipid metabolism, and regulation of gut dysbiosis. Future research should include further in-depth studies on strain identification, scale-up of postbiotics, identification of underlying mechanisms, and well-defined clinical studies. Postbiotics could be a promising dietary intervention for the prevention and management of obesity.

## 1. Introduction

Obesity is a growing public health problem, defined as a body mass index (BMI) of 30 kg/m^2^ or more, and is characterised by an unhealthy build-up of fat due to an energy imbalance in the adipose tissue of the body [1]. The increased fat accumulation gradually stimulates the unbalanced use and storage of energy, leading to dysfunction in glucose, protein, and lipid metabolism [2]. Obesity is a complex disease that is influenced by physiological, environmental, and genetic factors [3]. In addition, routine habits, including consumption of high-calorie foods and a sedentary lifestyle, contribute significantly to obesity. Moreover, there is increasing evidence that intestinal microbiota dysbiosis plays a crucial role in the prevalence of obesity [4,5]. Obese people may be more susceptible to oxidative stress. Thus, there is a relationship between oxidative stress, obesity, and gut microbiota [6].

Obesity is associated with a wide range of serious negative health effects, promoting the incidence of many comorbid conditions, such as hypertension, type 2 diabetes, nonalcoholic fatty liver disease, musculoskeletal disorders, cardiovascular diseases, and certain cancers [7,8]. Currently, obesity is prevalent in almost all age groups, and its global prevalence has nearly tripled between 1975 and 2016 [9]. In 2019, the global numbers of obese and overweight adults were 650 million and 1.9 billion, respectively [10]. According to estimates, by 2025, one in five adults worldwide will be obese, and the obesity prevalence in men and women will be 18% and 21%, respectively [11]. Another major concern is rising childhood obesity, which is most prevalent in the US [12]. Statistical analysis revealed that 330 million children and youth between the ages of 5 and 19 and 40 million children under 5 years of age were overweight or obese in 2016 [13]. Despite the fact that South Koreans are leaner than Westerners, obesity concerns have grown there as well. In South Korea, obesity is defined as a BMI of ≥25.0 kg/m^2^. Obesity prevalence has increased in South Korea from 31.3% in 2009 to 37.1% in 2021 [14]. Moreover, it is anticipated to gradually rise to 62% in men and 37% in women by 2030 [15]. Class III obesity (≥35.0 kg/m^2^) has increased in both Korean men and women, according to South Korean definitions of obesity that follow World Health Organization recommendations for the Asia–Pacific region [16]. Thus, more effective methods for the prevention and treatment of obesity are required in the near future.

Adipose tissue is categorised as white adipose tissue (WAT) and brown adipose tissue (BAT), which differ in their morphology and anatomical position, development patterns, and metabolic functions [17]. WAT stores energy in the form of triglycerides, produces adipokines, and acts as a vital endocrine organ. WAT adipocytes are characterised by large lipid droplets, peripheral nuclei, and a few mitochondria [18]. In contrast, the characteristic features of BAT adipocytes include multiple small lipid droplets (multilocular), an oval central nucleus, and many mitochondria with uncoupling protein-1 (UCP-1) expressed in their inner membrane. With the help of UCP-1, BAT plays an active role in energy expenditure by distributing chemical energy as heat, or non-shivering thermogenesis. This process regulates body temperature and provides protection against obesity by increasing energy expenditure [19]. Individuals with obesity and diabetes have lower BAT size and activity than individuals with a normal BMI [20]. A third type of thermogenic adipocyte, named beige or ‘brite’, develops in WAT in response to various stimuli. It is characterised by small lipid droplets and mitochondria, and appear after stimulation such as cold exposure [21]. It shares a common origin with WAT, with similar morphology and functions [22]. An increased number of beige adipocytes in WAT due to either de novo adipogenesis or transdifferentiation from mature WAT is a common phenomenon known as browning, beiging, and brittening [23]. Beige adipocytes are abundant in humans, making them a prospective treatment option for obesity and other related diseases. Current research suggests that several transcriptional regulators and co-regulators that affect BAT adipocyte development also play important roles in transforming WAT adipocytes into beige adipocytes [24,25]. For instance, factors required by brown adipocytes, including PR domain-containing 16 (PRDM16), peroxisome proliferator-activated receptor γ (PPARγ), UCP-1, and peroxisome proliferator-activated receptor gamma coactivator-1 alpha (PGC-1α), have been found to be the primary targets for WAT transdifferentiation [23,24]. PRDM16 promotes the development of brown and beige adipocytes and upsurges the expression of genes related to WAT in mice and human fibroblast cells in vitro, in addition to increasing the expression of PPARα and PGC-1c, which are important for maintaining brown fat. Increased expression of PRDM16 leads to increased beige and brown adipogenesis in WAT. PGC-1α is a PPARγ coactivator that is required to initiate thermogenesis by promoting the transcription of UCP-1 [19]. UCP-1 is a widely studied marker that is expressed in BAT and beige adipocytes. In contrast, fatty acid-binding protein 4 (FABP4) and fatty acid synthase (FAS) are involved in the development of mature adipocytes; PPARγ and CCAAT/enhancer-binding protein α (C/EBPα) are reported to be the main activators of adipogenesis [26,27].

## 2. Probiotics

Probiotics are among the most explored and utilized functional food ingredients, with several health-promoting properties [28]. Probiotics are defined as “live microorganisms that when administered in adequate amounts, confer a health benefit on the host” [29]. However, in probiotic-containing supplements (components), the relative proportions of active and non-active probiotic cells may differ significantly, and the number of non-active cells may surpass that of active cells. Therefore, the positive benefits of supplements may be related to the presence of non-viable components (postbiotics) in probiotic-containing products [30,31]. Probiotics have been shown to exert anti-obesity effects by suppressing oxidative stress and endoplasmic reticulum stress, regulating lipid, cholesterol, and glucose metabolism, changing gut microbiota composition, and reducing chronic low-grade inflammation markers and cytokines [32]. Moreover, the anti-obesity effects of probiotics are strain-, genus-, and species-specific and are based on a mixture of probiotic species [33]. Furthermore, studies have revealed differences in the anti-obesity effects mediated by live and lyophilised lactic acid bacteria (LAB) [34].

Despite the health-promoting effects of probiotics, including anti-obesity effects, studies on probiotics have underlined their limitations, such as strain-specific actions, unidentified molecular mechanisms, short-lived mechanisms, niche-specific action, presence of antibiotic resistance genes and their transfer, virulence-related gene transfer, haemolytic activity (alpha and beta), degradation of intestinal mucosal layer, ambiguous beneficial effects, production of biogenic amines (food spoilage and food safety concerns), production of D-lactic acid (induction of acidosis), hindering the establishment of commensal gut microflora, capacity to induce opportunistic infections (bacterial translocation across the epithelial layer), occurrence of harmful metabolic enzymes (β-glucuronidase, nitroreductase), inflammatory response, infective endocarditis, sepsis, bacteraemia in immunocompromised individuals, and limitations for use in industry, including lack of viability and functionality in the manufacturing process and alteration in the flavour and aroma of probiotic products, are significant bottleneck issues [35,36,37,38]. Thus, it is essential to look for alternative strategies, such as the use of postbiotics. These soluble products could exert similar health benefits on the host as live probiotic cells.

## 3. Postbiotics

The word “postbiotic” comes from the Greek words “post” (after) and “bios” (life). The gastrointestinal (GI) tract of humans is a distinctive, intricate microbial system, harbouring trillions of viruses, bacteria, fungi, and archaea [39,40]. Gut bacteria influence several host physiological activities, including complex mutual interactions with the host immune system [41] and acquiring nutrients required for their growth from the host [42]. During their life cycle, bacteria secrete low-molecular-weight metabolites that are essential for controlling their own development, growth, and propagation, as well as for stimulating the growth of other useful organisms, promoting cell-to-cell communication, and protecting against environmental stresses [43,44,45]. Some of these soluble mediators may be produced by living bacteria or liberated after bacterial lysis into surroundings, offering further functional advantages by altering cellular activities and biochemical functions [42]. Postbiotics are bioactive components (products or metabolic by-products) that are either produced by viable bacteria or liberated after bacterial lysis, which may have a helpful effect on the host [42].

According to the International Scientific Association for Probiotics and Prebiotics (ISAPP), postbiotics are “the preparation of non-viable microorganisms and/or components that provide health benefits to the host”. This recent definition blends the terms postbiotics and paraprobiotics [46]. ISAPP also stressed that postbiotics must include microbial cells or cellular factors that have been attenuated with or without metabolites, and that have been shown to have positive effects on health. It is necessary to characterize a preparation’s microbial composition prior to attenuation in order to consider it postbiotic. Therefore, it has been proposed that postbiotics can be distinguished as microbial factors made from foodstuffs fermented by known microorganisms rather than customary foods fermented by unknown microorganism cultures [46]. Different researchers refer to postbiotics by several names, including ‘abiotic’, ‘biogenic’, ‘cell-free supernatant’, ‘ghost probiotic’, ‘metabiotic’, ‘paraprobiotic’, ‘postbiotic’, and ‘pseudoprobiotic’. Postbiotics are commonly regarded as the non-viable portions of probiotic cells [31].

Prebiotics have also been extensively studied and shown to have anti-obesity benefits by modifying gut microbiota, according to research [47]. Recently, it has been shown that phenolic compound fractions alone or in combination with polysaccharides can modify gut microbiota [48,49]. Hence, prebiotics with postbiotics should also be considered as a potential strategy for the prevention and treatment of obesity.

### Production and Characterisation Approaches

Postbiotic production methods can be categorised into natural and laboratory methods. One of the most significant sources of naturally produced postbiotics is the fermentation process. During fermentation, microbial cells use prebiotic substances, naturally or in response to external factors, produce a variety of postbiotic compounds with different biological properties, including antimicrobial, antioxidant, and anticancer properties, which in turn results in the improvement of the food matrix with these useful substances [50]. Approaches reported for producing postbiotics include heat inactivation, ionising radiation, formalin inactivation, ultraviolet (UV) rays, high pressure, dehydration, supercritical fluid technology (CO_2_), pH modification, sonication, and omics technologies [51,52]. Pulsed electric fields, ohmic heating (also known as electroconductive heating), and, most recently, high-intensity ultrasound (HIUS) have been demonstrated to inactivate probiotic bacteria [51,53]. These inactivation techniques are presently suitable for the bench-scale production of postbiotics. However, further research is needed to create innovative techniques for enhanced commercial scale-up of postbiotic production that maintain functional benefits while being cost- and time-effective.

Potential reasons for bacterial cell viability loss include mechanical injury, denaturation of genetic material, shattered cell membranes, and changes in the physiological state of bacteria [54]. Future studies should focus on the identification and characterisation of novel postbiotics. The various analytical approaches used for the characterisation of probiotics include Raman spectroscopy, flow cytometry, atomic force microscopy, proton-based nuclear magnetic resonance, infrared spectroscopy, and gas and liquid chromatography tandem mass spectrometry [52].

Many probiotics are known to produce postbiotics, including the *Lactobacillus*, *Bifidobacterium*, *Streptococcus*, *Bacillus*, and *Faecalibacterium* genera [55]. A recent review documented the postbiotic ability of the probiotic yeast *Saccharomyces boulardii* [56]. The advantages of postbiotics include a lack of strict production or storage requirements while emulating the properties and actions of probiotics. Thus, there is no inconvenience to their use even in underdeveloped countries [57]. Regarding safety, postbiotics have the advantage of preventing the development of resistance and virulence genes, which may occur in vivo when utilising probiotics [58]. Furthermore, postbiotics minimize the requirement for exposure to live microbes, which is crucial for kids with developing immune systems and leaky intestinal barriers.

**Figure 1:**
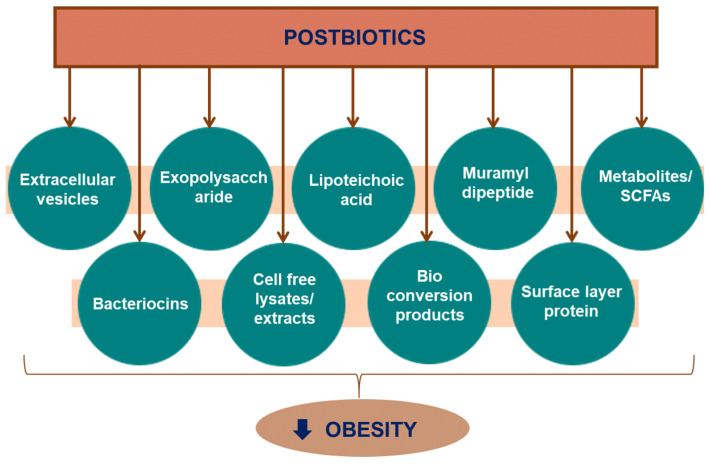
The present figure highlights different postbiotics evaluated against obesity. ↓ = Decrease.

A comprehensive review targeting the anti-obesity effects of postbiotics and their mechanisms has not been reported yet. Postbiotics exert anti-obesity effects via multiple mechanisms, with the major mechanisms including increased energy expenditure, reduced adipogenesis and adipocyte differentiation, suppression of food intake, inhibition of lipid absorption, regulation of lipid metabolism, and regulation of gut dysbiosis. Figure 1 demonstrates different important postbiotics that have been used for the prevention and treatment of obesity in in vitro, in vivo, and clinical studies.

## 4. Postbiotics and Anti-Obesity Mechanisms

### 4.1. Cell and Animal Modal Studies

#### 4.1.1. Cell Wall Components

**Figure 2:**
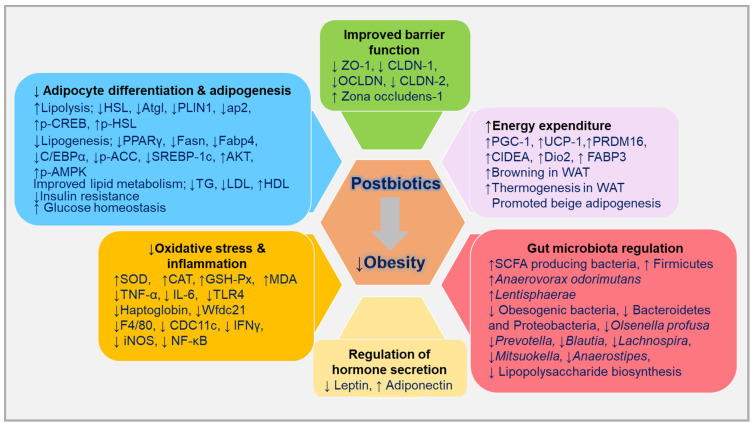
Different anti-obesity mechanisms of postbiotics in in vitro and in vivo models. ↓ = Decrease, ↑ = Increase.

Pattern recognition receptors (PRRs) are subdivided into two categories: cytoplasmic and membrane-bound. The cytoplasmic category includes nucleotide-binding oligomerisation domain (NOD)-like receptors (NLRs) and retinoic acid inducible gene I-like receptors (RLRs), while C-type lectin receptors (CLRs) and membrane-bound receptors include toll-like receptors (TLRs), which sense extracellular pathogens and are localised to the plasma membrane and endosomes [59]. NLRs comprise a large family of cytosolic sensory receptor proteins that are thought to play key roles in innate immune response and inflammation [60]. Among the cell wall components, lipoteichoic acid (LTA), muramyl dipeptide (MDP), exopolysaccharide (EPS), and S-layer proteins (SLPs) have been identified as major anti-obesity factors (Table 1). Various anti-obesity mechanisms of postbiotics have been presented in Figure 2.

##### Muramyl Dipeptide

Peptidoglycan is an important component of the bacterial cell wall, comprising specific muropeptide arrangements detected by NOD proteins. Muropeptides containing diaminopimelic acid (meso-DAP) and muramyl dipeptide (MDP) are recognised by NOD1 and NOD2, respectively [61]. MDP has been detected in both Gram-negative and Gram-positive bacteria. According to Cavallari et al. [62], MDP supplementation was a beneficial postbiotic that lowered adipose inflammation and glucose intolerance in obese mice by NOD2 and interferon regulatory factor 4 (IRF4, an estrogen-regulated gene) independent of weight loss or microbiome composition alteration. The authors also demonstrated that MDP reduces hepatic insulin resistance in diet-induced and hyperphagic obesity. Furthermore, IRF4 is not involved in the NOD1 signalling process [62]. The same research group later established the importance of activating receptor-interacting serine/threonine kinase 2 (RIPK2) for the protective effect of MDP. The stimulation of RIPK2 has been hypothesised to be the second molecular pathway underlying NOD2-mediated glucose tolerance improvement. These outcomes were found in non-haematopoietic cells, indicating that this mechanism is cell type-specific [63] (Table 1). RIPK2 is a downstream signalling molecule required by NOD1 and NOD2 [64].

The glucose-lowering actions of MDP and its dependence on adipocyte IRF4 have recently been identified as being sex-dependent. Researchers have shown that adipocyte IRF4 is essential for the blood glucose-lowering effects of MDP in male mice with endotoxaemia and high-fat diet (HFD)-induced obesity. Surprisingly, the researchers discovered that obese female AdipoIRF4fl/fl (Table 1) mice had reduced glucose levels after MDP treatment. Despite male AdipoIRF4fl/fl mice being resistant to NOD2-mediated glucose alterations during low-level endotoxaemia and obesity, the study found that both groups (WTfl/fl and AdipoIRF4fl/fl) displayed reduced expressions of inflammatory markers after MDP treatment [65]. These studies indicate that postbiotics (MDP) could potentially be substituted for drug candidates against obesity and related disorders.

##### Surface Layer Proteins

Surface layer protein (SLP) is another important cell wall component of microorganisms (bacteria and archaea) [66]. SLPs are glycoproteins that adhere to the host intestinal wall and account for 15% of the total cellular protein fraction [67]. SLPs are mostly attached to the peptidoglycan layer of the cell wall through noncovalent interactions [67].

Kim et al. [68] evaluated the protective properties of SLPs isolated from kefir probiotic LAB, *Lentilactobacillus kefiri* DH1 and DH5, and *Leuconostoc mesenteroides* LCM6, LCM8, and LCM9. SLPs were isolated, purified, and identified using electrospray ionisation quadrupole time-of-flight mass spectrometry (ESI Q-TOF-MS). SLPs significantly decreased the levels of inflammatory markers in LPS-stimulated RAW 264.7 cells. In the HFD-fed mouse model, SLPs targeted obesity and related metabolic disturbances by improving systemic inflammation, adipogenesis, and insulin resistance [68] (Table 1).

Recently, heat-killed *Lactobacillus curvatus* (HKLC), *Lactobacillus plantarum* (HKLP), and various SLPs (SLPs, LPSLP, and LCSLP) from plants (kimchi) have been shown to inhibit lipid accumulation in 3T3-L1 cells. Adipogenic expression was significantly reduced by HKLPs and SLPs. Notably, SLP administration induced apoptosis in 3T3-L1 cells. This study emphasises the preventive effects of plant-based postbiotics on obesity [69] (Table 1). Together, these findings highlight the multifactorial outcomes of SLPs as postbiotics.

##### Lipoteichoic Acid

Like the other two components described above, lipoteichoic acid (LTA) is also a part of the cell wall structure in Gram-positive bacteria that recognises PRRs and triggers a signalling cascade [70]. These macroamphiphilic postbiotic components exhibit multifactorial bioactivities [71,72]. Previous studies have reported immunomodulatory and anti-inflammatory properties of LTAs from *Lactobacillus* and other bacterial species [73,74]. Moreover, a recent report showed the fat-reducing effects of LTA from *Bifidobacterium animalis* subsp. *lactis* BPL1 using the nematode *Caenorhabditis elegans* as a pre-clinical model. LTA is recognised as a novel lipid modulator with the ability to reduce fat via the insulin-like signalling pathway (IGF-1) [75]. In *C. elegans* and humans, IGF-1 has been shown to regulate lipid metabolism, immunity, and ageing [76,77]. This study suggests that LTA may have potent therapeutic and/or preventive applications in metabolic syndrome- and diabetes-related disorders [75] (Table 1).

##### Exopolysaccharide

The glycocalyx is an exterior polysaccharide covering bacterial cells; it is termed a capsular polysaccharide when it is strongly associated with the bacterial cell surface through covalent bonds and an exopolysaccharide (EPS) when it is loosely bound to the surface or is released into the environment [78]. Previous studies showed that the EPS produced by LAB exhibited functional characteristics, such as antioxidant, anti-inflammatory, immunological modulation, antiviral, antimicrobial, antitumour, and anti-biofilm activities [79,80]. However, few studies have demonstrated the use of EPS for the treatment of obesity. For instance, Zhang et al. [81] revealed that EPS from *Lactobacillus rhamnosus* GG can reduce adipocyte function via TLR2 signalling. EPS reduced triacylglycerol (TAG) levels without causing inflammation in cells. In addition, EPS supplementation reduced fat pads, lowered liver and serum TAG levels, and downregulated inflammation in HFD-fed mice [81] (Table 1). Another study by Lee et al. [82] established that EPS inhibited the differentiation of immature cells into mature adipocytes by upregulating the AMP-activated protein kinase (AMPK) signalling pathway and by downregulating the expression of adiponectin and adipogenesis markers. Activated AMPK can inhibit fat deposition and induce WAT browning by downregulating acetyl-CoA carboxylase (ACC) expression and impeding gluconeogenesis. Additionally, EPS showed an anti-adipogenic effect at the initial stage of adipocyte differentiation [82] (Table 1).

##### Surface Layer Protein, Exopolysaccharide, and Prebiotics

Seo et al. evaluated the effect of cellular components, including SLP and EPS, as postbiotics from kefir LAB against HFD-induced obesity and gut microbiota dysbiosis. The observed anti-obesity effect was greater when combined with prebiotic grape seed flour. The combined treatment (postbiotics + prebiotics) significantly reduced adipose tissue weight gain, body weight, and serum triglyceride (TG) levels. Microarray analysis of epididymal adipose tissue showed that combined treatment downregulated genes involved in adipogenesis, adipocyte differentiation, autophagy, acute-phase response, immune response, inflammatory response, lipid metabolic process, lysosomal program, and angiogenesis, while anti-inflammatory genes were upregulated. Moreover, the combined treatment increased the abundance of Proteobacteria. Of note, the expression of Akp13, the A-kinase anchoring protein 13 gene, which is linked to BMI and immunological response, was adversely associated with the prevalence of short-chain fatty acid (SCFA)-producing and obesogenic colon bacteria [83] (Table 1). This study demonstrated that EPS, SLP, and prebiotics work synergistically to exert anti-obesity effects by improving HFD-induced chronic inflammation, adipogenesis, and glucose intolerance along with alteration of intestinal microbiota.

#### 4.1.2. Biotransformation Products

Recent studies have investigated the use of postbiotics as bioconversion products. Kefir is a traditional fermented beverage, and LAB from kefir milk have been shown to have anti-obesity effects in vivo [84]. The same research group developed highly bioactive postbiotics from the biotransformation of whey and citrus using LAB from kefir. The authors tested this bioconversion product in an HFD-fed mouse model and found that it significantly ameliorated body weight gain, the adipose tissue weight/body weight ratio, plasma TG concentration, and adipocyte size. Postbiotic products upregulated gene expression associated with energy expenditure in adipose tissue and were found to be associated with the conversion of hesperidin to hesperetin. The product also had a considerable effect on the butyrate-producing bacteria *Olsenella profusa* and *Anaerovorax odorimutans*. Moreover, PGC-1α expression was significantly correlated with obesogenic markers, whereas UCP-1 expression was significantly correlated with *A. odorimutans* levels [85] (Table 1).

#### 4.1.3. Cell-Free Extracts

Cell-free extracts have been evaluated for their beneficial roles [86]. Osman et al. [87] demonstrated that postbiotics with a high protein content (27.5% crude protein) regulated lipid metabolism and thus could be utilised as a safe alternative to anti-obesity and anti-dyslipidemic agents. A postbiotic generated from sonicated *Lactobacillus paracasei* was given to HFD-fed Wistar rats for 9 weeks to evaluate the impact on lipids and weight. The postbiotic group was compared to atorvastatin-treated or placebo-treated rats. Both atorvastatin and postbiotic prevented a surge in body weight and reduced serum triglycerides, total serum lipids, and total serum cholesterol [87] (Table 1).

Another study showed that culture supernatants referred to as “BS” (*Bifidobacterium longum* DS0950; *L. rhamnosus* DS0508B) could effectively prevent HFD-stimulated body weight gain in mice, alter the expression of thermogenesis-related genes, and contribute to macrophage polarisation. BS-mediated browning of adipose tissue altered energy metabolism and promoted thermogenesis via protein kinase A/cAMP response element binding protein (PKA/CREB) signalling in 3T3-L1 cells. This study showed that *L. rhamnosus* DS0508 (BS) promoted browning and lipolysis in in vitro and in vivo models [88] (Table 1). In continuous research, supernatants of *B. longum* DS0950 and *B. bifidum* DS0908, isolated from human faeces, were evaluated as anti-obesity agents in HFD-fed mice. Probiotics and supernatants reduced weight gain and fat accumulation without affecting food intake in HFD-fed mice. Culture supernatants promoted thermogenesis by activating PKA-p38 mitogen-activated protein kinase signalling in C3H10T1/2 mesenchymal stem cells [89].

#### 4.1.4. Extracellular Vesicles

Extracellular vesicles (EVs) are spherical, microbially derived entities that are discharged into the extracellular environment and contain biomolecules such as proteins, polysaccharides, lipids, enzymes, and toxins [90]. According to reports, EVs affect immune responses in both healthy and pathological situations and play crucial roles in communication (inter- and intraspecific) [90,91].

Intestinal barrier dysfunction, such as a leaky barrier, is linked to obesity and other diseases [92]. *Akkermansia muciniphila* is a beneficial gut microbe that plays a crucial role in sustaining gut and metabolic health [93,94]. Serendipitously, pasteurising *A. muciniphila* improved its beneficial effects on obesity, glucose tolerance, and insulin resistance in obese and diabetic mice [95]. Furthermore, EVs from *A. muciniphila* showed improved efficacy against obesity. EVs derived from *A. muciniphila* triggered a more significant loss in body weight and fat content of HFD-given mice than the bacterium itself [96]. Subsequent studies investigated the effects of EVs from live and pasteurised *A. muciniphila* on obesity and showed that all treatments inhibited obesity-promoting mechanisms by reducing HFD-induced adipose tissue accumulation and liver inflammation, and by upregulating the expression of homeostasis and lipid metabolism genes. Notably, beneficial effects were more noticeable with the inactive form of EVs than with the active form. Furthermore, all treatments improved gut dysbiosis by increasing beneficial microbiota and reducing pathogenic bacterial development [97] (Table 1). These findings suggest a potential preventive strategy involving EV and pasteurised *A. muciniphila* (a paraprobiotic agent).

#### 4.1.5. Metabolites

Urolithins are naturally present bioactive substances formed after intense gut microbial activity on ellagitannin (hexahydroxydiphenoic acid esters) and ellagic acid (bilactone compound of hexahydroxydiphenic acid, found in plants as part of ellagitannin or as a glucoside) [98]. Following ingestion, ellagitannins are hydrolysed in the gut to ellagic acid, which then undergoes a series of metabolic reactions (hydrolysis, decarboxylation, and dehydroxylation) by the gut microbiota to produce urolithins (intermediates), such as urolithin C (Uro-C), urolithin A (Uro-A), urolithin D (Uro-D), and urolithin B (Uro-B) [98]. Among them, Uro-B and Uro-A are the key metabolites in the gut and are recognised as the most biologically active [99,100]. An in vivo study investigated the modulatory effects of Uro-A and Uro-B supplementation on gut dysbiosis in HFD-fed rats. Both intermediates independently modulated the relative abundance of microbial species related to body weight gain by increasing the abundance of *Parabacteroides* and decreasing the abundance of *Desulfovibrionacea* and *Coriobacteriaceae*, thus demonstrating anti-obesity properties [101]. The molecular rationale suggests that Uro-A induces WAT browning and increases thermogenesis in BAT, leading to increased energy consumption (thyroid hormone-dependent) [102] (Table 1).

SCFAs are important products produced by gut microbiota via the fermentation of non-digestible carbohydrates and have recently gained significant attention. Given their significance, several extensive reviews of their physiological features and pharmaceutical potential have recently been published [103,104,105,106]. Monocarboxylic acids with carbon atom counts under six are prevalent in the intestines and plasma. The three most significant SCFAs are butyrate, acetate, and propionate [107]. The relative quantities of each SCFA are influenced by the microbiota profile, with Firmicutes primarily generating butyrate and Bacteroidetes primarily producing acetate and propionate [108]. SCFAs exert interactive roles in the gut microbiota, energy metabolism, diet, and weight control [109]. Moreover, diet-induced thermogenesis has been reported to reduce obesity in both humans and animals [110]. Furthermore, the increase in energy expenditure induced by SCFA is linked to the lipid oxidation of the entire body, which also involves an increase in BAT activity [111,112].

For instance, Gao et al. [113] provided evidence of the thermogenic activity and therapeutic value of butyrate-enriched HFD-fed mouse models. Sodium butyrate supplementation increased fatty acid oxidation, thus increasing energy expenditure. In addition, enhanced mRNA expression of PGC-1α and UCP-1 (thermogenic markers) was reported in butyrate-treated mice. These results suggest that butyrate is resistant to obesity [113]. Lin et al. [114] provided molecular insights into the preventive effects of SCFA on diet-induced obesity in mice by demonstrating that butyrate and propionate, but not acetate, could stimulate the release of gut hormones and decrease food consumption [114] (Table 1).

Another study provided the first evidence that butyrate (oral but not intravenous administration) improved energy metabolism via the gut–brain neural circuit in a cholesteryl ester transfer protein (CETP) mouse model (a translational model for diet-induced obesity akin to that in humans). Butyrate-treated mice showed anti-obesity effects by reducing food intake, improving plasma lipid metabolism, increasing fat oxidation (during the day), and increasing the thermogenic activity of BAT tissues. Further research has revealed that butyrate can modify the gut flora without affecting the vagus nerve [115] (Table 1). Studies have shown the favourable effects of butyrate on energy metabolism and suggested that oral treatment might be an effective approach for combatting obesity and other metabolic disorders.

Recent studies have claimed that acetate stimulates BAT activity and promotes the formation of beige adipocytes [112,116,117]. Hanatani et al. described the involvement of acetate in browning using in vitro and in vivo models. In 3T3-L1 cells, acetate supplementation increased the expression of browning markers, and similar results were reported in vivo [112] (Table 1). Another study reported that acetate supplementation in drinking water increased the expression of thermogenic markers and induced mitochondrial biogenesis in BAT in a mouse model. This study also highlighted the role of G-protein-coupled receptor (GPR) 43 activation in brown adipocytes, which promoted mitochondrial biogenesis and enhanced the energy expansion capacity of cells. Moreover, acetate induced adipogenesis and mitochondrial biogenesis in immortalised brown adipocyte cells (IM-BAT) [116] (Table 1). Another study reported the induction of browning markers by acetate in an HFD-fed mouse model [117] (Table 1). Inhibition of chronic inflammation and modification of bacterial dysbiosis by an increase in the proportion of Bacteroidetes and a decrease in the proportion of Firmicutes have also been considered as preventive measures against obesity in HFD-fed mice [118] (Table 1).

In addition to SCFAs, metabolites produced after the digestion of anthocyanins by the gut microbiota also play an important role in the induction of thermogenesis. For instance, vanillic acid, which is created by the intestinal microbiota through the metabolism of anthocyanins, induced thermogenesis in BAT and WAT browning after 16 weeks in high-fat-/high-sucrose-diet-induced obese mice [119]. Similarly, ketoA (10-oxo-12(Z)-octadecenoic acid), another putative postbiotic, increased energy expenditure. KetoA is a derivative of linoleic acid generated by LAB in the gut that can activate the sympathetic nervous system by activating the ion channel transient receptor potential vanilloid 1 in the GI tract, thereby increasing energy expenditure through the stimulation of BAT activity and inguinal WAT browning [120].

#### 4.1.6. Bacteriocins

Bacteriocins are produced by several LAB strains and are widely used in the food sector as postbiotics. These are ribosomally synthesised antimicrobial peptides with adequate heat stability and safety [121]. A few studies have evaluated the role of bacteriocins as anti-obesity agents. The bacteriocin plantaricin EF system produced by *L. plantarum* reduced body weight and food intake in HFD-fed mice. However, no effect was observed on the gut microbiota composition [122]. Another study reported gut microflora modulation as an alternative strategy using PJ4 bacteriocin produced by *Lactobacillus helveticus* PJ4 for the treatment of obesity. In accordance with these results, PJ4 suppressed body weight gain by reducing adipocyte size [123] (Table 1).

#### 4.1.7. Cell-Free Lysates

An investigation of the association between gut microbiota and obesity identified obesogenic and obesity-preventive bacterial species. Firmicutes, Bacteroidetes *Lactococcus*, *Rhizobium*, and *Clostridium* are examples of obesogenic gut microbiota [124]. The genus *Lactobacillus* has long been identified as beneficial against obesity. In recent years, the question of whether the modulation of gut dysbiosis through postbiotic supplementation could be a novel strategy to treat obesity has garnered significant attention from the scientific community. In this regard, the use of heat-killed *Ligilactobacillus salivarius* strain 189 (HK LS 189) supplementation as an anti-obesity and gut microbiota modulator has been recently documented. The effect of postbiotic supplementation was investigated for four weeks in a pig model of obesity, which showed a significant inhibitory effect on growth, an increase in the fraction of *Parabacteroides*, and a decrease in the *Prevotella* proportion. Beta analysis showed significant differences in microbial composition (Firmicutes, Bacteroidetes, and Proteobacteria); the abundance at the phylum level was 87.2% in the heat-killed lactobacilli supplemented group compared to that in the control group (97.7%). Notably, pathways related to lipid metabolism, metabolism, the excretory system, and signal transduction were significantly raised in the HK LS 189 group. Moreover, the study also described functional pathway analysis, which suggests that metabolism and lipid metabolism are significantly different between the two groups [125] (Table 1).

Previously, it was demonstrated that the heat-killed *Lactobacillus* strain (*L. plantarum* OLL2712) regulated blood glucose metabolism by controlling inflammatory cytokine expression [126]. Similarly, a recent study demonstrated the ameliorating effects of heat-treated *L. plantarum* HK L-137 against obesity and adipose tissue inflammation in an animal model, highlighting that HK L-137 supplementation reduced plasma levels of lipopolysaccharide-binding protein (LBP), a marker of intestinal permeability, in HFD-fed mice. Overall, the study found that HK L-137 reduced adipose tissue inflammation, transient weight gain, and liver damage, at least in part by increasing intestinal permeability and decreasing endotoxin translocation [127] (Table 1).

**Table 1:** Effects of postbiotics on obesity parameters: in vitro and in vivo studies.

Source	Postbiotic	Model	Treatment/Duration	Biological Effects	Reference
**Muramyl dipeptide**					
Commercial	MDP	Mouse: wild type (WT) C57BL/6J, Irf4^−/−^ mice, hepatocyte-specific NOD2^−/−^ mice, and leptin-deficient (ob/ob) mice	(a) MDP 100 µg; 4 days/week, 5 weeks), + HFD. (b) HFD, 10 weeks + daily injection of MDP, 3 days + final MDP injection before 24 h of experiments	↓Obesity, ↓hepatic insulin resistance, ↓fat inflammation Upregulated NOD2-IRF4 pathway	Cavallari et al. [63]
Commercial	MDP	Male and female: Adipocyte-specific *Irf*4^−/−^ (IRF4 knockout mice (AdipoIRF4^fl/fl^) and control without adiponectin-cre transgene (WT^fl/fl^), 3 days before LPS injection + 6 h fasting	Acute endotoxaemia experiments: MDP 100 μg, 3 days, 4th day, LPS (i.p., ultrapure) 0.2 mg/kg, 6 h prior to GTT. Diet-induced obesity (a) Standard chow diet (b) Treatment group (60% kcal fat diet) + MDP (100 μg) 4 days/week, GTT performed At 5th week, eWAT collected	MDP: For blood glucose-lowering effects during endotoxaemia and HFD-fed male mice, adipocyte IRF4 was essential. No alteration in glucose level in endotoxaemia in female mice HFD-fed female mice had lower blood glucose level than the control. Inflammatory markers: ↓TNF, ↓CCL2, ↓CXCL10, ↓CD8, ↓IL-1β, ↓IL-10, ↓IL-6, ↓IFNγ, ↓CD4, ↓NOS2, and ↓NLRP3 in male and female mice in both groups	Duggan et al. [65]
**S-layer protein**					
*Lentilactobacillus kefiri* (DH1 and DH5 (SDH1 and SDH5) and *Leuconostoc mesenteroides* DH1606, DH1608, and DH1609 (LCM6, LCM8, and LCM9)	SLPs	RAW 264.7 cells	LPS (0.1 μg/mL), SLPs (10 or 20 μg/mL), 24 h at 37 °C	↓IL-6 in LCM8, LCM9 and SDH5 ↓NF-κB p65 with SLCM9	Kim E. et al. [68]
		C57BL/6J (*n* = 10/group)	(a) Control group (saline) (b) Treatment HFD-SDH5 group (SLP from DH5 [SDH5] 120 mg/kg BW), and HFD-SLCM8 group (SLP from LCM8 120 mg/kg BW), 6 weeks	In SDH5 and SLCM8: ↓body weight gains and adipose tissue weight, ↓plasma TG, ↓insulin In SDH5: ↓TC, ↓LDL-C In SLCM8: adipocyte differentiation: ↓KLF8, acute phase response: ↓TRDN, ↓LBP, adipogenesis: ↓ADAM23, autophagy: ↓MAP1a, ↓Atp6v0d2, immune response: ↓ADAM8, ↓SLAMF7, ↓DCSTAMP, ↓MARC1, ↓UBD, ↓TREML4, ↓DOCK8, Inflammatory response: ↓EAR12, ↓HORMAD2, ↓NCAN, ↓Gpr50, ↓LIPF, ↓OXTR, ↓TREM2, ↓RGS1, ↓Tm4sf19, ↓Sfrp5 Upregulated gene expression ↑SH2B2, ↑MOGAT1, ↑FCNA, ↑EBF 2, ↑GPX3, ↑KLHL2, ↑CCR8, ↑CES1f/2c, ↑C2	
*Lactobacillus curvatus* (HKLC), and *Lactobacillus plantarum* (HKLP)	SLPs and heat-killed LAB	3T3-L1 preadipocytes	SLPs (LC and LP) 7.5 μg/mL, 48 h	Inhibited lipid accumulation: LPSLP (19%), LCSLP (24%), and LPCSLP (24%), ↓FABP4, ↓PPARγ,. Induced apoptosis: ↓BCL-2, ↑caspase 3, HKLC, HKLP, and HKLPC: ↓FABP4	Yoon et al. [69]
**Lipoteichoic acid**					
*Bifidobacterium animalis* subsp. *lactis* BPL1 (CECT 8145)	LTAs	*Caenorhabditis elegans*	LTA BPL1-0.1, 1.0, 10, 20, 50 μg/mL	↓Fat content in nematodes ↓TG through involvement of IGF-1 pathway	Balaguer et al. [75]
**EPS**					
*Lactobacillus rhamnosus* GG	EPS	3T3-L1 cells	10.0 μg/mL, 6 days	↓ Intracellular TAG, Genes of adipose differentiation and adipogenesis: ↓PPARγ, ↓SCD1, ↓ap2, ↓DGAT1 ↓FAS, ↓LPL	Zhang et al. [81]
		C57BL/6J mice (n = 6)	(a) Control (b) HFD+ EPS (50 mg/kg, every two days), 14 days	Significant ↓fat mass: subcutaneous, gonadal, and pararenal, ↓serum TAG levels, ↓TAG and cholesterol ester in liver, ↓PPARγ, ↓ap2, ↓FAS, ↓SCD1, ↓LPL, ↓DGAT1.	
*Lactobacillus plantarum* L-14	EPS	3T3-L1 cells and hBM-MSCs	50 and 100 μg/mL	L-14 extract inhibited differentiation of 3T3-L1 cells and hBM-MSCs. ↓TAG storage in mature adipocytes ↓PPARγ, ↓C/EBPα, ↓FAS, ↓LPL, ↓CD36, ↓GPDH, EPS: ↑AMPK pathway ↓PPARγ, ↓C/EBPα, ↑adiponectin, ↓p-ACC, ↓SREBP-1c, ↑AKT, ↑p-AMPKα	Lee et al. [82]
**Surface layer protein, exopolysaccharide, and prebiotics**					
Kefir LAB: *Leuconostoc mesenteroides* DH 1606 (LCM6) and *L. mesenteroides* DH 1608 (LCM8)	EPS S-layer protein	C57BL/6 mice (*n* = 120)	(a) Control: HFD + saline (b) HFD + LCM6 EPS (250 mg/kg BW) (c) HFD + LCM8 SLP (120 mg/kg BW) (d) HFD-fed 2% wine grape seed flour (GSF) (e) HFD + EPS (42 mg/kg BW) + SLP (20 mg/kg BW) + GSF (0.5%) (all), 6 weeks	↓Body weight gain: SLP (20%) and all (23%) ↓Adipose tissue weight: SLP (22%) and all (24%). ↓Plasma TG EPS (16%), SLP (31%), 2% GSF (18%), and all (34%) EPS: ↓LDL-C (34%), ↓TC/HDL ratio (18%), and ↑HDL-C (10%) ↓ Area under the curve (AUC) for insulin response: SLP (22%), 2% GSF (14%), and all (23%) ↓Glucose response (AUC): all (14%) Acute-phase response: ↓TRDN, ↓LBP, Differentiation of adipocyte: ↓KLF8, Adipogenesis: ↓ADAM23, ↓BMP3, ↓POSTN, Autophagy: ↓CLEC4A3, ↓PLD4, ↑GSDMC, ↑HK2, Immune response: ↓S100A8, ↓DOCK8, ↓EAR10, ↓MPEG1, ↓UBD, ↓AKAP13, Lysosomal program: ↓LIPA, Inflammatory response: ↓PTAFR, ↓FGF13, ↓OXTR, ↓KNG1, ↓SERPINA3M, Lipid metabolism: ↓NCEH1, Anti-inflammation: ↑USP2, angiogenesis: ↓SERPINF1, ↓ANGPTL4, ↑SCFA-producing bacteria, ↓Obesogenic bacteria	Seo et al. [83]
**Biotransformation products**					
*Lentilactobacillus kefiri* DH5 (LKDH5) from Kefir	CPB (postbiotic) from whey (WHE) and citrus pomace extract (CPX)	C57BL/6J	(a) Control group (b) HFD + whey group (WHE) (c) HFD + LKDH5 group (108 CFU/kg BW; LAB) (d) HFD + citrus pomace extract group (10 mL/kg BW; CPX) HFD + postbiotics group (10 mL/kg BW; CPB)	↑Hesperetin, CPB group: ↓adipose tissue weight/body weight ratio, ↓TG Adipose tissue: ↑UCP-1, ↑PGC-1α ↑*Anaerovorax odorimutans* ↓*Olsenella profusa*	Youn et al. [85]
**Cell-free extract**					
*Lactobacillus paracasei*	CFE	Wistar albino male rats	(a) Control basal diet (b) HFD group (c) ATOR group: HFD+ ATOR (10 mg/kg) (d) Treatment: HFD+ CFE-1 (100 mg/kg) (e) HFD+ CFE-2 (200 mg/kg), 9 weeks	↓Body weight gain, ↓serum lipid level CFE-1 (29%), and CFE-2 (34%), ↓TG level CFE-1 (32%), and CFE-2 (45%), ↓serum cholesterol CFE-1 (32%), CFE-2 (39%), ↓LDL-C CFE-1 (38%), CFE-2 (50%), ↑HDL-C CFE-1 (20%), CFE-2 (30%) ↓MDA, ↑SOD, ↑CAT, and ↑GSH-px.	Osman et al. [87]
*Bifidobacterium longum* DS0956 and *Lactobacillus rhamnosus* DS0508 28 = *Bifidobacterium longum* DS0950;13 = *Bifidobacterium bifidum* DS0908; 30 = *B. longum* DS0956; 51 = *Lactobacillus rhamnosus* DS0508	CFE	3T3-L1 preadipocytes	1, 5, or 10 μL per 1 mL	Brown-adipocyte markers: ↑PGC-1α, ↑UCP-1, and ↑PRDM16, beige-cell genes: ↑CD137, ↑FGF21, ↑P2RX5, and ↑Tbx1, white markers: ↑ap2, ↑PPARγ. Lipolysis factor: ↑HSL, ↑ATGL Activated PKA pathway ↑p-CREB, ↑p-HSL Reversed downregulated expressions of UCP-1, PGC-1α, and PPARγ	Hossain et al. [88]
*BSs = 30 = B. longum* DS0956*; 51 = Lactobacillus rhamnosus* DS0508		C57BL/6N mice (*n* = 56)	(a) Control: ND + saline (b) model control: HFD + saline (c) HFD + MRS broth (150 μL) (d) HFD + BS30 (150 μL) (e) HFD +BS51 (150 μL) (f) HFD+ BS30(10^9^ CFU/kg BW) (g) HFD+BS51(10^9^ CFU/kg BW), 12 weeks	↓Body weight: BS groups, ↑UCP-1, PGC-1α, PRDM16 (BS 30), ↑HSL, ↑PLIN1, ↑aP2. (BS 30) M1 polarisation marker: ↓IL-1β, ↓TNF-α (BS 30) M2 polarisation marker: ↑ARG1, ↑CD206 (BS 30)	
*B. bifidum DS0908*, *B. longum DS0950*	CFE	Male C57BL/6 mice (*n* = 56)	(a) Group1: NFD (b) Group2: HFD (c) Group3: HFD + culture supernatants of DS0908 (150 mL/model) (d) Group 4: HFD + culture supernatants of DS0950 (150 mL/model) (e) Group 5: HFD + DS0908 bacterial pellet (1 × 10^9^ cells/kg) (f) Group 6: HFD + DS0950 bacterial pellet (1 × 10^9^ cells/kg) (g) Group 7: HFD + Rosiglitazone (10 mg/kg), 8 weeks	↓weight gain ↓fat accumulation, improved insulin sensitivity and glucose metabolism. Lipid profile, Culture supernatant: ↓TG Both pellet and supernantant: ↓LDL, ↓cholesterol, ↑HDL	Rahman et al. [89]
		C3H10T1/2 MSCs	5 μL per 1 mL	↑UCP-1, ↑PPARγ, ↑PGC-1α Beige adipocyte-specific markers: ↑P2RX5, ↑FGF21, Brown adipocyte-specific marker: ↑Cox2 ↑p-PKA,↑p-p38 MAPK, ↓AMP, ↓CREB S133	
**EVs**					
*Akkermansia muciniphila*	Evs	Male C57BL/6 mice	First group: (a) HFD + PBS (control, 200 mL) (b) HFD + *A. muciniphila* alive 10^9^ CFU/200 μL, (c) HFD + eVs (10 μg protein/200 μL), 5 weeks Second group: (d) ND + PBS (control, 200 mL) (e) ND + *A. muciniphila* alive 10^9^ CFU/200 μL, (f) ND + eVs (10 μg protein/200 μL), 5 weeks	Both treatments: low level of body weight gain and substantial reduction in food intake in HFD mice ↓Adipocyte size in both treatments but more noticeable in eVs. eVs group: ↓TC, ↑PPARα, ↑PPARγ, ↓TGF-β Alleviated inflammation: ↓TLR-4, ↓TNF-α, ↓IL-6. Improved barrier function: ↑CLDN-1, ↑ZO-1, ↑OCLDN, and ↓CLDN-2. Both treatments influenced body weight in the ND group.	Ashrafian et al. [96]
		Caco-2	10 μg of EV, 24 h	EV: ↑ZO-1, ↑OCLDN, and ↑CLDN-1	
*Akkermansia muciniphila* Live and pasteurised (P)	eVs	Male C57BL/6 mice	(a) Control: ND + PBS 200 μL (b) Control: HFD + PBS 200 μL (c) Treatment: HFD + 10^9^ CFU/200 μL *A. muciniphila* (Live) (d) HFD + 10^9^ CFU/200 μL *A. muciniphila* (P) (e) HFD + 10 μg protein/200 μL eVs, 5 weeks	↓Food intake in pasteurised bacterium and its eVs EV: ↓adipocyte size and showed normal morphology ↓TG, ↓LDL, ↑HDL. ↓TNF-α, ↓IL-6, ↑IL-10 ↑ZO-1 and ↑OCLDN (highest in EV), ↑CLDN-1 (highest in P), ↓CLDN-2 (EV and P). Colonic inflammation markers: ↑TLR-2, ↑IL-10, ↓TNF-α, ↓TLR-4 (EV highest) Lipid metabolism: ↑Angptl4 (highest in EV)	Ashrafian et al. [97]
**Metabolites**					
Commercial	Urolithin A (Uro-A), urolithin B (Uro-B)	Wistar rats (*n* = 24)	(a) Control group (normal diet) (b) HFD weekly (c) HFD+ Uro-A (2.5 mg/kg) (d) HFD+ Uro-B (2.5 mg/kg), 4 times/week, 4 weeks	Significant reduction in final body weight ↓Cholesterol, ↓LDL-C, ↓TG, ↑HDL-C Uro-A: ↑Bacteroidetes, ↑Proteobacteria, Restored Firmicutes Uro-B: ↑Firmicutes, ↓Proteobacteria	Abdulrahman et al. [101]
Pomegranate ellagitannin (eTs)	Uro-A	C57BL/6 mice, leptin-deficient ob/ob mice	(a) ob/ob mice normal diet, (b) C57BL/6 mice on HFD (c) UA (30 mg/kg/day) (d) Orlistat (15 mg/kg/day) (e) Vehicle (0.1% Tween 80) by gavage, daily, 6–10 weeks	UA prevented both diet-induced and genetic obesity ↓Adipocytes, ↓fat mass, ↓body weight, ↓Plasma TG ↑Energy expenditure, ↑Thermogenesis in BAT, ↑browning in WAT In liver: ↓TNF-α, ↓IL-6, ↓TG, ↓liver weight	Xia et al. [102]
SCFA	Butyrate	Male C57BL/6J mice	(a) Control group (b) HFD + Na butyrate (5% *w*/*w*), 12 weeks	↑Fatty acid oxidation, ↑thermogenic markers (PGC-1α and UCP-1) in BAT ↓adiposity, ↑mitochondrial biogenesis and function in BAT	Gao et al. [113]
SCFA	Butyrate, Acetate, and Propionate	Male C57BL/6N mice	(a) Control group (b) HFD + Na salts of butyrate (5% *w*/*w*) (c) HFD+ acetate (3.7% *w*/*w*) (d) HFD+ propionate (4.3% *w*/*w*), 4 weeks	Butyrate and propionate inhibited weight gain completely, acetate 40% suppression Butyrate and propionate reduced food intake and stimulated gut hormones	Lin et al. [114]
SCFA	Butyrate	Male APOE*3-Leiden.CETP (E3L.CETP)	(a) Control group (b) HFD + Na butyrate (5% *w*/*w*), 9 weeks	Prevented body weight gain and weight of the g-WAT, ↑Fat oxidation, ↑thermogenic marker (UCP-1) in BAT	Li et al. [115]
SCFA	Acetate	3T3-L1 cells	Na acetate (1 mM), 3 days	↑UCP-1, ↑PGC-1α, ↑PRDM16, ↑PPARα, ↑DiO2, ↑CIDEA, ↑FABP3 Beige adipocyte-selective genes: ↑TMEM26, ↑TBX1	Hanatani et al. [112]
		Male KK-Ay mice (obese diabetic)	(a) Control group (b) Treatment group: sodium acetate (0.6%, oral supplementation), 16 weeks,	↑Thermogenic markers in BAT ↑Browning markers in WAT	
SCFA	Acetate	Immortalised BAT	Acetate (10 mM) or acute treatment 6 h (10 mM)	↑PPARγ, ↑AP2, ↑PGC-1α, ↑UCP-1 ↑p-ERK1/2, ↑p-CREB, ↑GPR3	Hu et al. [116]
		Male C57BL/6J mice	Na acetate (150 mM), 6 weeks, in drinking water	↑PGC-1α, ↑UCP-1	
SCFA	Acetate	Male C57BL/6JRj mice	(a) Low-fat diet (b) HFD + SCFA (5%) (c) HFD + acetate: propionate (10:1) (d) HFD + acetate: propionate (1:2.5), 30 weeks	Acetate suppressed hepatic lipogenesis ↑Body temperature ↑Browning markers’ expression in WAT	Weitkunat et al. [117]
SCFA	Acetate, Propionate, Butyrate, and their admixture	Male C57BL/6 J mice	(a) Control group (b) HFD group (c) HFD + Na acetate (d) HFD + sodium propionate (e) HFD+ sodium butyrate (f) HFD+ admixture (3:1:1 ratio), 16 weeks	Inhibited body weight gain Biochemical parameters: ↓TG, ↓cholesterol, ↓IL-1β, ↓MCP-1, ↓IL-6 ↑GPR43 expression in the adipose tissue and decreased expression in colon ↓Leptin expression by acetate or SCFA admixture ↑Expression of adiponectin and resistin by all SCFAs or their admixture Promoted beige adipogenesis	Lu et al. [118]
Metabolite of anthocyanins	Vanillic acid	Male C57BL/6N mice	HF/high sucrose diet + vanillic acid (0.5%), 16 weeks	↑Expression of browning markers, and thermogenic markers, ↑cold tolerance, ↑mitochondriogeneis in BAT and WAT	Han et al. [119]
Linoleic acid metabolites	KetoA	Male C57BL/6N mice	HF diet + KetoA (0.;1%), 10 weeks	↑UCP-1 in BAT and WAT ↑Expression of thermogenic markers and browning markers Activation of TRPV1 and SNS	Kim et al. [120]
**Bacteriocins**					
Bacteriocin	Plantaricin EF	Male C57BL6/J mice	(a) HFD (b) HFD+ 2 × 10^9^ cells of *L. plantarum* NICMB8826-R (20 μL) (LP group) (c) HFD+ 2 × 10^9^ cells of LM0419 (20 μL), 9 weeks (MU group)	Reduced food intake and weight gain. No change in gut microbiota (LP group). ↑Zonula Occludens-1 in LP group	Heeney et al. [122]
Bacteriocin	PJ4 by *L. helveticus* PJ4	Male C57BL/6 J mice	(a) Control group (b) HED group (c) HFD + DT24 (d) HFD + TSU4 (e) HFD + PJ4 (H + P), 50 μL/mL/animal/day, 30 days	Group: PJ4 more promising results: Significant decrease in body weight, ↓Adipocyte size, ↓TC, ↓TG, ↓LPS, ↓insulin, ↓IL-1β, ↓IL17, ↓IL-6, ↓IL-10, ↓IL14, ↓IFNγ, ↓TNFα, ↓MCP-1, ↓Adipokine and inflammasome. Modulated gut microflora: ↑Firmicutes, ↓Bacteroidetes and Proteobacteria	Bai et al. [123]
**Cell-free lysates**					
*Ligilactobacillus salivarius* strain 189	Heat-killed (HK LS)	Pigs (*n* = 48)	Control group: a corn-soybean meal (CON), a basal diet Treatment group: a basal diet supplemented with HK LS 189 (0.2%), 4 weeks	Significant reduction in final body weight and daily weight gain. Significant difference in β-diversity between two groups. ↑Lentisphaerae, ↓*Prevotella*, ↓*Blautia*, ↓*Lachnospira*, *YS2*_ unclassified, ↓*Mitsuokella*, ↓*Anaerostipes*	Ryu et al. [125]
*Lactobacillus plantarum* L-137	Heat-killed (HK L-137)	C57BL/6 J (*n* = 30–32/group)	Three groups by body weight: (a) Normal group (64% as carbohydrates, 20% as protein, and 6% of energy as fat) (b) HFD group (62% of energy as fat, 18% as protein, and 20% as carbohydrates) + HK L-137 (0.002%) (c) HFD group without treatment, 4 to 20 weeks	↓Weight gain Plasma: ↓cholesterol, ↓glucose, ↓AST, ↓ALT level ↓LBP (a marker of endotoxaemia) plasma levels In eWAT epididymal adipose tissue: ↓CD11c, ↓IL-1β, ↓F4/80, ↓TNF-α, ↓MCP-1	Yoshitake et al. [127]
*Bifidobacterium longum* BR-108	Heat treated (IBL)	Male C57BL/6J mice	(a) Normal diet (b) HFD (c) HFD + IBL, 200 mg/kg BW, (d) HFD+ IBL, 400 mg/kg BW, 4 weeks	Reduced both weight gain and epididymal body fat mass, ↓TC, ↓cholesterol, ↓glucose, ↓LPS, ↓hepatic TG	Kikuchi et al. [128]
*Bifidobacterium longum* BR-108	Heat treated (IBL)	Male Tsumura Suzuki obese diabetes (TSOD) mice, genetically obese mouse	(a) Control: TSOD group (b) Control: Tsumura Suzuki non-obese (TSNO) group (c) TSOD + IBL (50 mg/kg) (d) TSOD + IBL (100 mg/kg) (e) TSOD + IBL (150 mg/kg), 30 days	Reduced the body weight gain 100 (6.5 g) and 150 mg/kg (7.2 g) Reduced adipose tissue buildup ↓blood glucose levels, ↓TC, ↓cholesterol, ↓FFA	Othman and Sakamoto, [129]
Kefir-derived lactic acid bacteria (LAB) + prebiotic LAB: *Leuconostoc mesenteroides* 4 (LMDH4), *Lactobacillus kefiri* DH5 (LKDH5)	Heat-killed HLAB	Male C57BL/6J	(a) Control group (HF and high-fructose diet (HFFrD) + microcrystalline cellulose 5% (b) HFFrD + GSF (2.5%) (c) HFFrD + HLAB (d) HFFrD+ GSF + HLAB (GSF+HLAB) 10 mL/kg BW, 8 weeks	Reduced body weight gain and adipose tissue weight gain ↓Haptoglobin (HP) ↓WFDC21 ↓FABP4 ↓FAS	Seo et al. [130]
*Lactobacillus plantarum* K8	LAB-P	3T3-L1 preadipocytes	(a) Control (b) LAB-P (12.5, 25, 50, 100, 200, 400 and 800 μg/mL), 2, 4, and 6 days	Suppressed lipid accumulation 50 (12%), 100 (42%), and 200 (58%) μg/mL. Reduced fat droplets ↓PPARγ, ↓C/EBPα, ↓FABP4 ↓p-JAK2, ↓p-STAT3 and ↓p-STAT5, ↑p-AMPKα	Kim H. et al. [131]
*Lactiplantibacillus plantarum* K8	LAB-P	Male C57BL/6J mice	(a) Normal group (b) Control (HFD group) (c) HFD + LAB-P (50 mg/kg BW/day) (d) HFD + LAB-P (100 mg/kg BW/day), 14 days	Reductions in weight gain Reduced HFD-induced hypertrophy: eWAT (36%), mWAT (20%), and iWAT (40%) ↓Hepatic fat accumulation, ↑p-AMPKα. Regulation of macrophages (adipose tissues): ↑CD206, ↓CD11c. ↓IL-1β, ↓IL-6, ↓NF-κB	Lim et al. [132]
*LactiplantibacillusPlantarum* (LP) K8	Heat-treated LPK8 (K8HK)	Male C57BL/6 mice	(a) HFD, 10 weeks (b) Live and heat-killed 10^9^ CFU/mL 2 weeks before start of HFD, 12 weeks	↓TG in both groups (live and heat-killed) ↓PPARγ, ↓C/EBPα, ↓FABP4	Jang et al. [133]
		3T3-L1 cells	Live and heat-killed 10^9^ CFU/mL	No cytotoxicity with heat-killed ↓TG, ↓PPARγ, ↓C/EBPα, ↓FABP4, ↓ACC, ↓FAS, ↓SCD1, ↑SOCS-1, ↓p-JAK2, ↓p-STAT3 compared to control	
*Lactobacillus brevis* KB290	Heat-killed KB290 (KB)	Male C57BL/6J mice	(a) NFD group (b) HFD group (c) HFD + KB, 2% (*w*/*w*), 8 weeks	↓Weights of epididymal and renal adipose tissue, ↓area of epididymal adipocytes ↑Adiponectin, ↑β3-adrenergic receptor In epididymal adipose tissue serum ↑FAA Altered microbiota composition	Watanabe et al. [134]

HFD, high-fat diet; AST, aspartate aminotransferase; ALT, alanine aminotransferase; LBP, lipopolysaccharide-binding protein; CD11c, cluster of differentiation 11c; IL-1β, interleukin-1β; TNF, tumor necrosis factor; MCP-1, monocyte chemoattractant protein-1; MDP, muramyl dipeptide; NOD2, nucleotide-binding oligomerisation domain-containing protein 2; IRF4, interferon regulatory factor 4; LPS, lipopolysaccharide; GTT, glucose tolerance test; CCL2, chemokine (C-C motif) ligand 2; CXCL10, C-X-C motif chemokine ligand 10; IL-6, interleukin 6; IL-10, interleukin 10; IFNγ, interferon gamma; CD4, cluster of differentiation 4; CD8, cluster of differentiation 8; NLRP3, NLR family pyrin domain containing 3; NOS2, nitric oxide synthase-2; SLP, surface layer protein; TG, triglyceride; TC, total cholesterol; LDL-C, low-density lipoprotein cholesterol; KLF8, Krüppel-like factor 8; TRDN, triadin; ADAM23, ADAM metallopeptidase domain 23; MAP1a, microtubule-associated protein 1A; Atp6v0d2, ATPase H+ transporting V0 subunit D2; ADAM8, a disintegrin and metallopeptidase domain 8; DOCK8, dedicator of cytokinesis 8; SLAMF7, SLAM family member 7 (the surface antigen CD319); DCSTAMP, dendrocyte expressed seven transmembrane protein; MARC1, mitochondrial amidoxime reducing component 1; UBD, ubiquitin D; TREML4, triggering receptor expressed on myeloid cells like 4; EAR12, eosinophil-associated, ribonuclease A family, member 12; HORMAD2, HORMA domain containing 2; NCAN, neurocan; Gpr50, G-protein-coupled receptor; LIPF, lipase, gastric; OXTR, oxytocin receptor; TREM2, triggering receptor expressed on myeloid cells 2; RGS1, regulator of G-protein signalling; Tm4sf19, transmembrane 4Lsix family member 19; Sfrp5, secreted frizzled-related sequence protein 5; SH2B2, SH2B adaptor protein 2Acsm3; MOGAT1, monoacylglycerol O-acyltransferase 1; FCNA, ficolin A; EBF 2, early B cell factor 2; GPX3, glutathione peroxidase 3; KLHL2, Kelch-like 2; CCR8, chemokine (C-C motif) receptor 8; CES1f/2c, carboxylesterase 1F/2c; C2, complement component 2 (within H-2S); LAB, lactic acid bacteria; FABP4, fatty acid–binding protein 4; PPARγ, peroxisome proliferator-activated receptor-γ; BCL-2, B-cell lymphoma-2; LTAs, lipoteichoic acids; IGF-1, insulin-like growth factor-1; HDL-C, high-density lipoprotein cholesterol; Uro-A, urolithin A; Uro-B, urolithin B; UCP-1, uncoupling protein 1; PGC-1α, peroxisome proliferator-activated receptor gamma coactivator 1-alpha; AMPK, AMP-activated protein kinase; C/EBPα, CCAAT enhancer binding protein α; FAS, fatty acid synthase; LPL, lipoprotein lipase; GPDH, glycerol-3-phosphate dehydrogenase; EPS, exopolysaccharide; p-ACC, phosphorylated acetyl-CoA carboxylase; p-AMPKα, phosphorylated-AMP-activated protein kinase alpha; SREBP-1c, sterol regulatory element-binding transcription factor 1; AKT, protein kinase B; Angptl4, angiopoietin-like 4; SCFAs, short-chain fatty acids; ATOR, anti-dyslipidemia agent replacing atorvastatin; CFE, cell-free extract; MDA, malondialdehyde; SOD, superoxide dismutase; CAT, catalase; GSH-px, glutathione peroxidase; PRDM16, PR domain containing 16; FGF21, fibroblast growth factor 21; P2RX5, purinergic receptor P2X 5; TBX1, T-box transcription factor 1; aP2, adipocyte protein 2; HSL, hormone-sensitive lipase; ATGL, adipose triglyceride lipase; PKA, protein kinase A; p-CREB phosphorylated cAMP-responsive element binding protein; p-HSL, phosphorylated HSL; MAPK, mitogen-activated protein kinase; MRS, De Man, Rogosa and Sharpe; PLIN1, perilipin 1; ARG1, arginase 1; CD206, cluster of differentiation 206; EVs, extracellular vesicles; PBS, phosphate buffered saline; PPARα, peroxisome proliferator-activated receptor alpha; TGF-β, transforming growth factor beta; TLR-4/-2, toll-like receptor -4/-2; ZO-1, zonula occludens-1; OCLDN, occludin; CLDN-1/-2, claudin -1/-2; BAT, brown adipose tissue; WAT, white adipose tissue; TRPV1, transient receptor potential vanilloid 1; SNS, sympathetic nervous system; DiO2, iodothyronine deiodinase 2; CIDEA, cell death-inducing DNA fragmentation factor-α like effector A; FABP3, fatty acid binding protein 3; TMEM26, transmembrane protein 26; p-ERK1/2, phosphorylated extracellular signal-regulated kinases 1 and 2; GPR43, G-protein-coupled receptor 43; FFA, free fatty acids; WFDC, whey-acidic protein four-disulfide core domain; p-JAK2, phosphorylated Janus kinase 2; p-STAT3/5, phosphorylated signal transducer and activator of transcription factor 3/5; eWAT, epididymal white adipose tissue; mWAT, mesenteric WAT; iWAT, inguinal WAT; NF-κB, nuclear factor kappa-light-chain-enhancer of activated B cells; SCD1, stearoyl-CoA desaturase 1; SOCS-1, suppressor of cytokine signalling-1. ↓ = Decrease, ↑ = Increase.

The anti-obesity effects of inactivated cells (heat-treated) of *B. longum* (BR-108) in HFD-fed mice [128] and genetically obese mice [129] were demonstrated by significantly lowering body weight gain, adipose tissue mass gain, and altered intestinal microflora (Table 1).

Treatment with heat-killed paraprobiotic kefir LAB (*L. mesenteroides* 4 [LMDH04] and *L. kefiri* DH5 [LKDH5]) significantly reduced weight gain and adipose tissue weight gain. In addition, a synergistic positive anti-obesity effect was observed after combination with prebiotics in Western-diet stimulated mice [130]. Cell lysates of *L. plantarum* K8 have lipid-lowering effects by activating AMPKα signalling and deactivating Janus kinase 2/signal transducer and activator of transcription 3/5 (JAK2-STAT3/5) signalling in vitro [131]. Another study demonstrated the ameliorating effects of heat-treated *Lactiplantibacillus plantarum* K8 on obesity and related inflammatory responses in vivo by suppressing the expression of adipogenic factors [132]. Another recent study evaluated the effects of heat-treated *Lactiplantibacillus plantarum* K8 in vitro and in vivo. Of note, the study reports that the heat-treated K8 at a concentration of 1 × 10^9^ CFU/mL demonstrated anti-obesity effects in contrast to its live counterpart, which displayed cytotoxicity. This study also reveals that heat-treated K8 acts on 3T3-L1 cells to upregulate the expression of negative regulators like suppressor of cytokine signalling-1 (SOCS-1). The JAK2/STAT3 pathway may be inhibited by SOCS-1 which would then suppress the expression of genes related to the development of obesity. In contrast to the in vitro study, both live and heat-treated cells showed weight reduction in the animal model [133].

Another study documented the anti-obesity effect of heat-killed *Lactobacillus brevis* KB290 in HFD-fed obese mice, indicating a paraprobiotic role for this bacterium. Mice in the paraprobiotic group showed a higher abundance of Bacteroides than that in the other groups [134] (Table 1).

### 4.2. Clinical Studies

The use of postbiotics to alleviate obesity has been validated in some human studies. In overweight individuals (BMI = 25–40 kg/m^2^), acute administration of inulin-propionate ester significantly increased the secretion of postprandial peptide tyrosine tyrosine (PYY, acts to lower appetite) and glucagon-like peptide-1 (GLP-1) and decreased calorie intake. Compared with the control, long-term intake resulted in a significant reduction in weight gain and intra-abdominal adipose tissue distribution. Long-term dosing also decreased the amount of lipids found within liver cells [135] (Table 2).

Van der Beek et al. [136] tested the effect of acetic acid (SCFA) administration in the distal colon of obese patients [BMI (mean) = 31.0 kg/m^2^]. Compared with the placebo group, an increase in fat oxidation and PYY concentration was observed in the treatment group [129] (Table 2). Canfora et al. [137] conducted a similar distal colonic dosing study of SCFA mixtures in 13 obese normoglycaemic men (BMI = 25–35 kg/m^2^). Compared with a placebo, colonic infusions of SCFA mixtures resulted in a significant increase in fat oxidation, resting energy expenditure, and PYY. Furthermore, SCFA supplementation decreased lipolysis in tested patients [137] (Table 2). Prebiotics are generally considered to be safe and to have negligible side effects. To a large extent, it is still unknown whether the derived metabolites are beneficial or harmful. As such, we do not know exactly the interplay between prebiotics and the diverse gut environment. Hence, future studies should concentrate on the safety of materials obtained through various treatments of prebiotics.

Inspired by their study on the anti-obesity effects of *Pediococcus pentosaceus* LP28 in diet-fed obese mice [138], Higashikawa et al. tested the paraprobiotic LP28 (from *P. pentosaceus*) in overweight patients. Overweight patients (*n* = 62) were administered LP28 (live), a placebo, or heat-killed LP28 powder orally once a day for 12 weeks. Supplementation with heat-killed LP28 led to a significant decrease in body fat percentage, waist circumference, BMI, and body fat mass compared to those with placebo [139] (Table 2). Nakamura et al. [140] evaluated the effect of heat-inactivated and lyophilised bacterial (*Lactobacillus amylovorus* CP1563) powder on lipid metabolism in 200 individuals with a BMI of 25–30 kg/m^2^ for 12 weeks. A daily dose of 200 mg of bacterial powder was provided as a water-based drink (500 mL). At the end of the intervention, heat-treated bacterial supplementation significantly reduced whole-body fat, body fat percentage, and visceral fat in the test group compared with those in the placebo group. Furthermore, significant improvements were observed in the anthropometric measurements and markers of glucose and lipid metabolism. No side effects were observed after the intervention [140]. The group also identified 10-hydroxyoctadecanoic acid (10-HOA) as the main constituent for obesity prevention in diet-induced obese mice. Sugawara and colleagues [141] reported the preventive effects of CP1563 in healthy pre-obese individuals (25.0–29.9 kg/m^2^) and its paraprobiotic effects on gut microbiota. After 12-week supplementation, the CP1563 group showed significant reductions in the areas of total, abdominal, visceral, and subcutaneous fat. In addition, paraprobiotics affect the gut microbiota through significantly higher changes in *Roseburia* and *Lachnospiraceae* and significantly lower changes in *Collinsella* compared to those by placebo [141] (Table 2). It is apparent that only a small number of clinical trials have looked at how postbiotics, mostly SCFAs and heat-killed probiotics, might help fight obesity. Thus, more clinical studies will be required in the future. To better comprehend the modulation and biochemical mechanism of postbiotics on metabolic activity, disease prevention, and maintaining human health, additional intervention studies involving metabolomics analysis ought to be carried out. High-tech advances have made it feasible to use metabolomics to examine the effects of probiotics, prebiotics, and postbiotics on gut health.

**Table 2:** Effects of postbiotics and paraprobiotics in obese and overweight patients.

Source	Postbiotic/Paraprobiotic	Subjects Type and Count (*n*)	Design/Duration	Format/Dose	Outcomes	Country/Reference
SCFA	Propionate	Overweight adults, 60	Randomised, double-blind, placebo-controlled, parallel design	Inulin-propionate ester (10 g/day) or inulin-control group (10 g/day), 24 weeks	↓weight gain, ↓intrahepatocellular lipid content, ↓intra-abdominal adipose tissue distribution, Inhibited decline in insulin sensitivity ↑PYY, ↑GLP-1	UK, Chambers et al. [135]
SCFA	Acetate	Overweight/obese men, 10	Randomised, double-blind, crossover trial	Distal and proximal colonic sodium acetate infusions (one each) colonic acetate (100 or 180 mmol/L dissolved in saline 120 mL) Placebo: 120 mL, 3 days	Distal colonic acetate: ↑Fasting fat oxidation. ↑PYY, ↑fasting circulating acetate, ↑postprandial glucose, ↑insulin concentrations, ↓TNF-α Proximal colonic acetate: no significant difference	Netherlands, van der Beek et al. [136]
SCFA	Acetate, butyrate, propionate	Overweight/obese men, 13	Randomised, double-blind, crossover study	HA: Na acetate (24 mmol 60%), Na propionate (8 mmol, 20%), Na butyrate (8 mmol, 20%). HP: Na acetate (18 mmol, 45%), Na propionate (14 mmol, 35%), Na butyrate (8 mmol, 20%). HB: Na acetate (18 mmol 45%), Na butyrate (14 mmol, 35%), Na propionate (8 mmol, 20%), all in 200 mL water. Placebo: 40 mmol sodium chloride in 200 mL water, 4 days	All treatments: ↑fasting fat oxidation. ↑PYY (fasting and postprandial plasma) ↓lipolysis HA and HP: ↑resting energy expenditure	Netherlands, Canfora et al. [137]
*Pediococcus pentosaceus* LP28 from longan fruit	Heat-killed LP28	Overweight, 62: heat-killed LP28: *n* = 21, placebo: *n* = 20, LP28 (living): *n* = 21	Randomised, double-blind, placebo-controlled, 12 weeks	LP28 (living) group: 10 mL spoon (10^11^ cells) Heat-killed LP28: 7.5 mL (10^11^ cells) Placebo: 7.5 mL	Heat-killed LP28: ↓body fat mass, ↓BMI, ↓waist circumference, ↓body fat percentages	Japan, Higashikawa et al. [139]
*Lactobacillus amylovorus CP1563* from human fecal samples	Fragmented CP1563	Overweight and mildly obese, 200 Test: *n* = 100, placebo: *n* = 100	Double-blinded, placebo-controlled, randomised clinical trial (RCT), 12 weeks	Beverages with fragmented CP1563: 200 mg in a 500 mL bottle per patient per day	↓Body fat percentage, ↓whole body fat, ↓visceral fat, ↓TG, ↓TC, ↓LDL-C, ↓diastolic blood pressure	Japan, Nakamura et al. [140]
*Lactobacillus amylovorus* CP1563	Fragmented CP1563 with 10-HOA	Healthy subjects, 109 Test: *n* = 100, placebo: *n* = 100	Randomised, double-blind, placebo-controlled, parallel study, 12 weeks	Beverages with fragmented CP1563: 200 mg (1.44 mg of 10-HOA) in a 500 mL bottle per patient per day	↓Abdominal fat, ↓total fat, ↓visceral fat, ↓subcutaneous fat ↑genera *Roseburia* and *Lachnospiraceae*	Japan, Sugawara et al. [141]

Abbreviations: SCFA, short-chain fatty acid; PYY, peptide YY; GLP-1, glucagon-like peptide-1; TNF-α, tumor necrosis factor-alpha; BMI, body mass index; TG, triglycerides; TC, total cholesterol; LDL-C, low-density lipoprotein cholesterol. ↓ = Decrease, ↑ = Increase.

## 5. Conclusions and Future Implications

**Figure 3:**
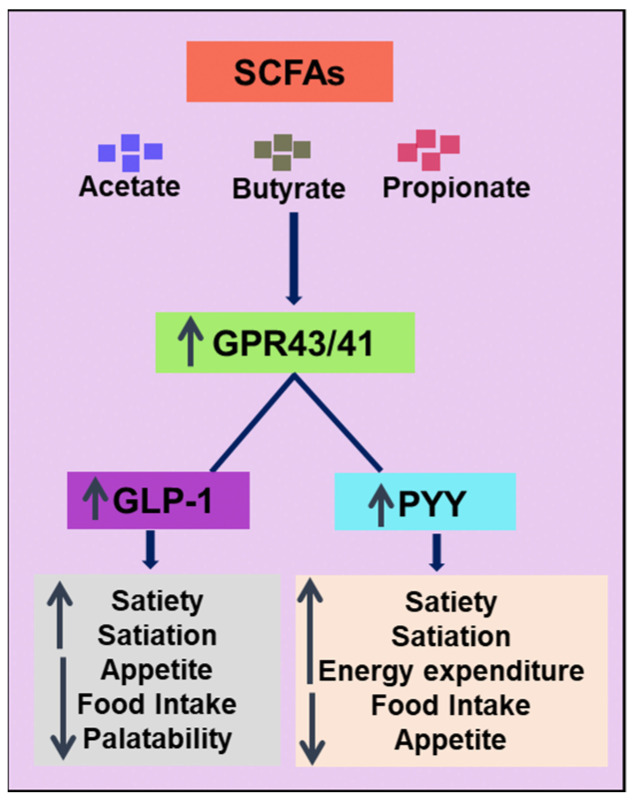
Anti-obesity effect of short-chain fatty acids (SCFAs). SCFAs activate G-protein-coupled receptors GPR-43 and GPR-41 that increase secretion of hormones, including glucagon-like peptide-1 (GLP-1) and peptide YY (PYY), manifesting anti-obesity effects. ↓ = Decrease, ↑ = Increase.

The frequency of obesity and related metabolic dysfunction has dramatically increased over the last few decades. The preventive and therapeutic effects of probiotics have been established. Probiotics are still used therapeutically; however, several drawbacks have not been fully addressed. Postbiotics, in contrast, are new dietary interventions, particularly for the prevention and management of obesity. The information presented in this review points to the possibility that postbiotics and paraprobiotics can alter biological responses to obesity in cell cultures, animals, and human volunteers. The effects of different metabolites from lysates and cell wall/membrane components from friendly bacteria are discussed in this review. The majority of these ingredients include MDP, SLP, LTA, EPS, urolithins, EVs, SCFAs, bacteriocins, and cell-free lysates. Most studies have been validated in animal models, including rats, mice, pigs, and nematodes, and a few randomised, controlled, double-blind clinical studies (RCTs) have focused on the use of SCFAs and heat-killed probiotic bacteria in overweight and obese individuals. Mechanistic studies using cell models have suggested that the anti-obesity effects of postbiotics are related to the upregulation of genes related to thermogenesis, browning, lipid oxidation, and catabolism. Reduced lipid storage in mature adipocytes and reduced expression of PPARγ, C/EBPα, adiponectin, phosphorylated ACC, and sterol regulatory element-binding transcription factor 1c (SREBP-1c) upregulated the AMPK signalling pathway. Moreover, the expression of interleukin 6 (IL-6) and nuclear factor-κB (NF-κB) was downregulated, and apoptosis was induced. In animal studies, postbiotic treatment boosted fatty acid oxidation, raised thermogenic biomarkers, increased browning marker expression, decreased food intake and weight gain, and reduced lipid accumulation by modulating signalling pathways and their downstream signalling molecules. Clinical studies have shown that postbiotic therapy decreases weight gain and boosts energy expenditure and lipolysis. The release of the intestinal hormones GLP-1 and PYY is enhanced by the activation of the receptors GRP-41 and GRP-43 by SCFAs, which ultimately causes patients to lose weight [142,143] (Figure 3).

More research is necessary to completely comprehend the mechanisms underlying these advantages in the prevention of obesity. In addition, more RCTs are needed to determine their potential value in the treatment and prevention of overweight and obesity, especially considering the dose and safety aspects. Only one study has evaluated the effect of fragmented Lactobacillus spp. on healthy individuals and reported that it modulates the composition of healthy bacteria. Thus, future studies will require an in-depth analysis of the gut microbiota profile in overweight and obese individuals. Compared to probiotics, postbiotics have several benefits, including a longer shelf life, simpler storage, and a lower need for low-temperature maintenance. From a safety perspective, postbiotics do not have the problem of antibiotic-resistance gene development due to probiotic virulence factors. Postbiotics have also been shown to exert indirect anti-obesity effects in vivo. A study discovered that postbiotics in resveratrol-fed mice may be the cause of the anti-obesity benefits of resveratrol. To develop deeper insights, future research should concentrate on further investigations, including clinical studies [144]. Overall, postbiotics created by LAB provide the potential for a constantly expanding functional food market as a component that can contribute to both value and innovation. It is crucial to optimise postbiotic production-related aspects, such as strain selection, media selection, and bioprocess setup, to produce postbiotics from LAB on an industrial scale, and future research should concentrate on locating and isolating multifunctional postbiotics. For newly discovered postbiotics, in silico analysis should be performed to examine their variable traits [145].

## Data Availability

Data derived from public domain resources.
